# Bile Biochemistry as a Therapeutic Target in Biliary Surgery: Disease-Specific Evidence and Clinical Limits

**DOI:** 10.3390/metabo16100721

**Published:** 2026-09-28

**Authors:** Zelimkhan Berikkhanov, Zakhar Akulov, Mihail Emcev, Evgeniy Tarabrin, Alexey Shestakov, Aleksey Kotelnikov, Andrey Nikolaev, Vadim Razumovsky, Milena Ivanova, Mehrshad Ebrahimnezhad, Sara Nourmahal, Sergey Muraviev

**Affiliations:** 1 Department of Hospital Surgery No. 2, Sechenov First Moscow State Medical University (Sechenov University), 119048 Moscow, Russia; berikkhanov_z_g@staff.sechenov.ru (Z.B.); emcev345@gmail.com (M.E.); tarabrin_e_a@staff.sechenov.ru (E.T.); shestakov_a_l@staff.sechenov.ru (A.S.); kotelnikov_a_g@staff.sechenov.ru (A.K.); nikolaev_a_m@staff.sechenov.ru (A.N.); ivanova_m_yu_1@staff.sechenov.ru (M.I.); nurmakhal_s@student.sechenov.ru (S.N.); muravev_s_yu@staff.sechenov.ru (S.M.); 2 Cardiology Department No. 2, Yudin City Hospital of Moscow, The Patrice Lumumba Peoples’ Friendship University of Russia (RUDN), 117198 Moscow, Russia; mehrshad.ebrahimnejad@gmail.com

**Keywords:** bile acids, enterohepatic circulation, cholestasis, cholelithiasis, FXR, bile acid transporters, biliary surgery

## Abstract

**Background/Objectives:** Bile acid therapies have expanded treatment options for cholestatic disease, but their relevance to biliary surgery depends on the diagnosis and endpoint. This critical narrative review examines where molecular evidence changes care and where surgical applications remain untested. **Methods:** The existing synthesis was supplemented by targeted literature searches and regulatory checks completed on 20 September 2026. Studies were assessed by design, population, outcome, and applicability to surgical decisions. **Results:** Genetic assessment can identify inherited cholestasis or selected recurrent lithogenic disorders that persist after anatomical treatment. Evidence for ileal bile acid transporter inhibitors supports indication-specific relief of cholestatic pruritus. Recent trials of receptor agonists show that biochemical, symptomatic, and disease-progression outcomes can diverge. Observational studies support biliary diversion in selected patients with inherited cholestasis, although comparisons among procedures and with drug treatment remain uncertain. The expanding genetic spectrum of progressive familial intrahepatic cholestasis and real-world treatment reports refine patient selection but provide limited evidence for long-term surgical outcomes. Bile metabolomics and synthesis markers remain unvalidated for timing cholecystectomy. **Conclusions:** Molecular findings are useful when they answer a defined diagnostic or therapeutic question. They can guide specialist treatment and surveillance alongside anatomical assessment. Prospective studies must establish whether molecular stratification improves outcomes in biliary surgical populations.

## 1. Introduction

Biliary surgery can remove stones, relieve obstruction, and treat gallbladder disease. It may leave an inherited defect in bile formation or a nonbiliary cause of symptoms unresolved. The practical question is whether knowledge of bile biochemistry changes a specific decision before or after an intervention.

Bile acids link cholesterol metabolism, intestinal absorption, microbial activity, and receptor signaling. Farnesoid X receptor (FXR) and the G protein-coupled bile acid receptor TGR5 regulate different aspects of bile acid homeostasis, inflammation, and metabolic function, with effects that depend on tissue and disease context [1,2]. Pathogenic variants in *ABCB11*, encoding the bile salt export pump BSEP, or *ABCB4*, encoding the phosphatidylcholine transporter MDR3, cause distinct cholestatic phenotypes [3]. These defined disorders differ from common multifactorial gallstone disease.

Existing reviews describe bile formation, lithogenesis, microbial bile acid metabolism, and pharmacological interruption of enterohepatic circulation [4,5,6,7]. For biliary surgery, the unresolved question is which findings change diagnosis, treatment selection, or the need for another intervention. Antipruritic efficacy in a child with inherited cholestasis, for example, cannot establish prevention of recurrent common bile duct stones in an adult. This review therefore evaluates evidence at the level of the clinical decision it can support.

We examine molecular diagnosis in recurrent or atypical biliary disease, disease-specific drug treatment, and non-transplant operations for inherited cholestasis. We separate measured clinical benefit from proposed perioperative applications and identify outcomes that require prospective study. The diagnostic uncertainty described as “asymptomatic cholecystitis” in a chapter by two of the present authors provides additional clinical context [8]. It is considered an author-proposed concept, not a validated indication for molecular testing or surgery.

## 2. Materials and Methods

This is a critical narrative review. The initial literature search covered PubMed/MEDLINE, Web of Science, Scopus, and the Cochrane Library using terms for bile acid metabolism, cholestasis, cholelithiasis, transporters, FXR, TGR5, enterohepatic circulation, and the gut–liver axis. During revision, targeted searches of indexed publications using Europe PMC, which includes PubMed/MEDLINE records, together with reference lists and publisher websites were completed on 20 September 2026. These combined disease and intervention terms, including “PFIC” and “biliary diversion”, “maralixibat” and “real-world”, and individual transporter genes or drug names. Official product information and withdrawal notices were checked separately.

The update prioritized 2025–2026 publications and earlier primary studies needed to interpret mechanisms or treatment effects. No uniform publication-year cutoff was imposed. Randomized trials and disease-specific comparative studies were preferred for efficacy claims. Observational cohorts and case series were included when they addressed rare genotypes, routine clinical use, or surgical procedures lacking trials. Guidelines supplied clinical context. Predominantly English-language publications were used, with relevant previously cited non-English material retained.

Selection was based on relevance to a mechanism, treatment, or diagnostic decision discussed in the review. Preclinical findings were identified explicitly. Conference summaries were not treated as definitive efficacy evidence. The sponsor-reported BOLD result was retained solely as a preliminary update on a negative postoperative outcome.

We compared study populations, controls, endpoints, follow-up, and applicability to biliary surgery. Regulatory approval was recorded separately from study design and with its jurisdiction specified. No formal certainty scores were assigned. The review had no registered protocol, duplicate independent screening, or pooled effect estimation and does not claim exhaustive retrieval.

## 3. Bile Metabolism in Health and Disease

Bile acids, phosphatidylcholine, cholesterol, and bilirubin affect different aspects of biliary disease. The following account retains the mechanisms needed to interpret cholestasis, lithogenesis, and the treatments discussed below.

### 3.1. Bile Acids

Cholesterol 7α-hydroxylase (CYP7A1) initiates the classical synthetic pathway. Rare loss-of-function variants in *CYP7A1* were associated with hypercholesterolemia and cholesterol gallstones in a family study [9]. This establishes a monogenic mechanism without making it a common explanation for gallstones. Serum 7α-hydroxy-4-cholesten-3-one (C4) reflects synthesis, while fibroblast growth factor 19 (FGF19) participates in feedback inhibition. Their evaluation in bile acid diarrhea does not validate either marker for postoperative cholestasis or selection for cholecystectomy [10].

CYP27A1 mediates sterol side-chain oxidation and initiates the alternative pathway, in which CYP7B1 contributes to chenodeoxycholic acid synthesis [11,12]. *CYP27A1* deficiency causes cerebrotendinous xanthomatosis, whereas *CYP7B1* deficiency can cause severe neonatal cholestasis [11,13]. Peroxisomal defects can also produce abnormal C27 intermediates, as documented in Zellweger syndrome [14]. These genotype-defined disorders show why atypical bile acid species and syndromic features may be more informative than the total bile acid concentration.

Conjugation with glycine or taurine by bile acid–CoA amino acid N-acyltransferase (BAAT) supports bile acid solubility and transport [1,15]. Ursodeoxycholic acid (UDCA) modifies the pool and can promote choleresis and cellular protection [16]. Neither this mechanism nor changes in transporter expression establish equal clinical benefit across all causes of cholestasis.

### 3.2. Secretion of Bile Acids and Inorganic Solutes

At the sinusoidal membrane, sodium-taurocholate cotransporting polypeptide (NTCP, encoded by *SLC10A1*) mediates sodium-dependent bile acid uptake, while organic anion-transporting polypeptides (OATPs) contribute sodium-independent transport [4,17,18,19]. During cholestasis, reduced uptake and increased basolateral export through organic solute transporter α/β (OSTα/β) and multidrug resistance-associated proteins 3 and 4 (MRP3/MRP4) can limit hepatocellular accumulation. High circulating bile acids may therefore reflect adaptive export as well as impaired biliary secretion. Concentration alone does not identify the cause.

NTCP-deficient mice were protected against obesity and hepatic steatosis [20], whereas human NTCP deficiency can present with conjugated hypercholanemia without comparably severe liver injury [21]. Species, substrate distribution, and the underlying disease constrain translation. NTCP also permits hepatitis B and D virus entry, providing a separate rationale for pharmacological inhibition [22,23].

Canalicular transporters have distinct substrates. BSEP (*ABCB11*) exports bile salts, multidrug resistance-associated protein 2 (MRP2, encoded by *ABCC2*) exports bilirubin conjugates and other organic anions, and MDR3 (*ABCB4*) supplies phosphatidylcholine to bile [19,24]. BSEP dysfunction causes a spectrum from recurrent cholestasis to severe progressive familial intrahepatic cholestasis (PFIC) [25,26]. MDR3 dysfunction produces phosphatidylcholine-poor bile and contributes to PFIC and low-phospholipid-associated cholelithiasis (LPAC) [3,27,28]. Restoring drainage alone may leave the abnormal bile composition unresolved.

PFIC extends beyond the classical *ATP8B1*, *ABCB11*, and *ABCB4* disorders [26,29]. Recent reviews describe at least 13 genetic forms involving transport, tight junctions, intracellular trafficking, receptor signaling, or ciliary function [30,31]. Evidence is uneven across these disorders. Residual BSEP function, extrahepatic disease, and liver disease stage are clinically more useful than assuming that every numbered subtype responds to the same treatment [3,32]. Their implications for surgery are considered in Section 4.4.

Aquaporins contribute to water movement during bile formation [33]. AQP8 expression was associated with steatosis in human samples, and increased expression improved steatosis through an FXR-related mechanism in obese mice [34]. The correction replaced a duplicated panel in Figure 2A of the cited study without changing the reported discussion or conclusions [34]. The study is retained as limited mechanistic evidence relevant to research on conservative treatment of biliary disease. It provides no clinical evidence for AQP8-directed treatment of cholestasis or cholelithiasis.

### 3.3. Biliary Phospholipids

MDR3-dependent phosphatidylcholine secretion protects biliary membranes and permits mixed micelle formation with bile salts and cholesterol [4,24,27]. Deficiency can favor both epithelial injury and cholesterol crystallization. Animal models support a causal link between impaired secretion and inflammation or fibrosis [35], but their severe phenotypes do not reproduce the full range of human disease.

Human genetic studies establish *ABCB4* deficiency as a cause of PFIC, while adult cohorts show presentations that include LPAC and other cholestatic phenotypes [36,37,38]. Genotype, residual function, and clinical presentation must be considered together. An isolated variant or altered transporter expression is insufficient to establish causality. Persistent intrahepatic stones or recurrent symptoms after cholecystectomy can justify specialist assessment for LPAC even when no pathogenic *ABCB4* variant is found [3].

### 3.4. Bile Pigments

Bilirubin handling illustrates why postoperative jaundice needs a cause-specific assessment. OATP1B1 participates in hepatic uptake, and MRP2 exports bilirubin conjugates [39,40]. Historical names such as OATP-C and SLC21A6 refer to OATP1B1, not additional transporters. MRP2 deficiency causes Dubin–Johnson syndrome, with conjugated hyperbilirubinemia rather than primary bile salt retention [40].

After obstruction is relieved, albumin-bound delta-bilirubin can slow the decline in measured direct bilirubin [41]. Hypoxia and inflammation can also alter transport, as experimental studies demonstrate [42]. Interpretation should combine symptoms, serial liver tests, and imaging [43]. New or worsening jaundice still requires assessment for obstruction, duct injury, infection, drug effects, and hepatic dysfunction.

### 3.5. Biotransformation and Enterohepatic Circulation

Intestinal microorganisms transform primary bile acids, including conversion of cholic acid to deoxycholic acid and chenodeoxycholic acid to lithocholic acid [44,45]. These reactions alter receptor activity and potential toxicity. Associations among dysbiosis, inflammatory bowel disease, and bile acid composition are affected by diet, inflammation, and treatment [6,46]. Experimental epithelial injury and carcinogenic signaling depend on the species and concentration studied [47]. Such findings identify mechanisms without yet defining a microbiota-based perioperative treatment.

The bile acid pool cycles repeatedly between liver and intestine. Most bile acids are recovered, and new synthesis replaces fecal loss [48]. Pool size and turnover vary with feeding, intestinal function, and disease, so fixed numerical estimates are not used as clinical thresholds here. The apical sodium-dependent bile acid transporter (ASBT, encoded by *SLC10A2*), also called the ileal bile acid transporter (IBAT), mediates active ileal uptake, and OSTα/β returns absorbed bile acids to portal blood [19,49,50]. ASBT and IBAT denote the same protein throughout this review.

Intestinal FXR (*NR1H4*) induces FGF19, which acts through hepatic FGF receptor 4 (FGFR4) and β-Klotho to suppress bile acid synthesis [1,5]. FXR also regulates transport and synthetic enzymes, including CYP8B1 [51]. TGR5 (*GPBAR1*) modulates inflammatory and metabolic responses through a separate receptor pathway [48,52]. Human tissue observations and experimental models link reduced biliary TGR5 expression to sclerosing cholangitis, but therapeutic efficacy in patients remains unproven [53].

Several related observations require separate interpretation. Fibroblast growth factor 21 (FGF21) findings in experimental fibrosis concern a different endocrine pathway [54]. FXR and FGF19 associations with hepatocarcinogenesis do not establish cancer-preventive treatment or a postoperative biomarker threshold [55,56]. Metabolic liver studies also concern a different population [57]. Current terminology is metabolic dysfunction-associated steatotic liver disease (MASLD) and metabolic dysfunction-associated steatohepatitis (MASH). The older terms nonalcoholic fatty liver disease (NAFLD) and nonalcoholic steatohepatitis (NASH) are retained for the original enrollment definitions of historical studies [58].

Table 1 links the principal molecular components to the evidence supporting their clinical interpretation.

## 4. Discussion

Therapeutic evidence is interpreted here according to its effect on a defined clinical decision. Symptom improvement, biochemical response, and native liver survival are assessed separately, since an intervention can succeed at one endpoint without improving another.

### 4.1. Molecular Targets and Emerging Therapies

Molecular diagnosis has the clearest clinical role in inherited cholestasis and selected recurrent lithogenic disorders, where it can direct disease-specific treatment and surveillance [3,63]. Scheme 1 summarizes this use of molecular information in patients with a defined clinical question.

#### 4.1.1. Established Treatment and Receptor-Directed Clinical Evidence

UDCA is an established treatment for primary biliary cholangitis (PBC) and is used in selected *ABCB4*-associated phenotypes, including LPAC [3,16]. These uses should not be conflated with universal treatment for postoperative cholestasis. In the PITCHES trial, UDCA did not significantly reduce the primary composite adverse perinatal outcome in intrahepatic cholestasis of pregnancy [64]. This result illustrates why benefit must be assessed for the particular disease and endpoint.

Obeticholic acid improved histological activity in the FLINT trial in noncirrhotic NASH, but pruritus and lipid changes complicated its benefit–risk profile [65,66]. The corrected FLINT report provides historical evidence for FXR modulation but did not evaluate biliary surgical outcomes. US approval of Ocaliva was withdrawn in November 2025 [67]. It is therefore not presented as a current US treatment option.

Newer FXR agonists have not produced uniform benefits. Tropifexor reduced liver fat and aminotransferases in a phase 2 NASH study, with dose-related pruritus [68]. The 2026 PRIMIS report provides a more direct test in primary sclerosing cholangitis (PSC). Among 419 randomized patients, cilofexor did not reduce fibrosis progression, and the trial was stopped for futility [69]. Biochemical target engagement cannot substitute for a demonstrated effect on disease progression.

Peroxisome proliferator-activated receptor (PPAR) agonists extend the treatment evidence beyond FXR. In PBC, the phase 3 RESPONSE trial found a biochemical response at 12 months in 61.7% of seladelpar recipients and 20.0% of placebo recipients. Pruritus also improved in patients with moderate-to-severe baseline symptoms [70]. In ELATIVE, elafibranor produced a biochemical response at week 52 in 51% versus 4%, but the key pruritus comparison was not significant [71]. Both received US accelerated approval in 2024 for adults with an inadequate response to UDCA or intolerance to it [72,73]. The approvals concern PBC and surrogate biochemical outcomes. They do not establish perioperative prophylaxis, reduced stone recurrence, or improved survival.

#### 4.1.2. IBAT Inhibitors in Clinical Trials and Routine Practice

IBAT inhibition reduces ileal reuptake and increases fecal bile acid loss. An earlier phase 2 maralixibat study in PBC did not show superiority over placebo for its primary pruritus endpoint [74]. Subsequent results demonstrate the importance of selecting the disease, agent, and endpoint. ICONIC supported an antipruritic effect of maralixibat in Alagille syndrome (ALGS), MARCH-PFIC demonstrated antipruritic benefit in a trial of 93 patients with PFIC, and PEDFIC 1 found reduced pruritus and serum bile acids with odevixibat in 62 patients with PFIC [75,76,77]. These trials provide disease-specific clinical evidence rather than a general indication for cholestasis.

US product information indicates maralixibat for cholestatic pruritus in Alagille syndrome from 3 months of age and in PFIC from 12 months. Odevixibat is indicated for pruritus in PFIC from 3 months and for cholestatic pruritus in Alagille syndrome from 12 months [78,79]. Formulation and weight restrictions must also be checked. Both labels identify limits in patients with specific *ABCB11* variants associated with absent or nonfunctional BSEP. Diarrhea, liver-test changes, and fat-soluble vitamin deficiency require monitoring.

Long-term evidence is encouraging but less secure than the short-term randomized findings. A comparison of maralixibat-treated Alagille trial participants with the GALA natural-history cohort suggested better event-free survival [80]. This was an external-control comparison, so residual confounding and differences in follow-up remain possible. It should not be described as randomized proof of transplant prevention.

Routine practice reports address patients not fully represented in registration trials. Himes and colleagues described eight patients with ALGS, including patients with previous biliary diversion or feeding-tube administration. Pruritus improved, although the uncontrolled design and small sample limit estimates of effectiveness and safety [81]. A later report described two patients with ALGS who received maralixibat before and after reversal of surgical biliary diversion [82]. These cases show that medical therapy can influence an individual surgical plan, but cannot establish when diversion should be reversed.

A prospective real-world cohort of 24 children treated with odevixibat included both classical and rarer PFIC genotypes and reported improvement in serum bile acids and pruritus over six months [83]. Short follow-up and genotype heterogeneity limit conclusions about transplant avoidance. In contrast, a 27-patient open-label maralixibat pilot in adult PSC was a clinical trial, not a real-world study. It found reductions in bile acids and improvement in some pruritus measures, with frequent diarrhea and no controlled assessment of disease progression [84].

A 2026 real-world case series reported improved pruritus in six patients with biliary atresia treated with maralixibat [85]. Biochemical responses were inconsistent, and progression to transplant listing or transplantation still occurred. This small uncontrolled experience supports further study of symptom management, not disease modification or routine use after the Kasai procedure.

In GLISTEN, 238 adults with PBC and moderate-to-severe pruritus were randomized to linerixibat or placebo. The adjusted between-group difference in monthly itch score over 24 weeks was −0.72 points (95% CI −1.15 to −0.28), favoring linerixibat [86]. Gastrointestinal adverse effects were frequent. US approval followed in March 2026 for cholestatic pruritus associated with PBC in adults [87]. The indication concerns itch relief, not gallstone prevention.

A clinically important negative result emerged from BOLD, which tested odevixibat after Kasai hepatoportoenterostomy in biliary atresia. The sponsor reported in July 2026 that the trial did not meet its primary endpoint of improved native liver survival [88]. This is a preliminary sponsor report pending full peer-reviewed results. It prevents an unqualified claim that IBAT inhibition improves outcomes across cholestatic disorders.

Elobixibat uses the same intestinal target to treat constipation in Japan, where increased colonic bile acid delivery is the intended effect [89,90]. Its indication illustrates why agents within this class cannot be treated as interchangeable. Across cholestatic indications, response should be judged alongside gastrointestinal tolerance, vitamin status, liver tests, and the outcome that prompted treatment.

#### 4.1.3. Transporter-Directed and Investigational Approaches

Selected BSEP trafficking defects may be pharmacologically rescuable. After in vitro evidence for 4-phenylbutyrate [91,92], a prospective uncontrolled study treated four patients with *ABCB11* missense variants and documented improved pruritus, bile acid concentrations, and canalicular BSEP expression [93]. This is limited human evidence for variant-dependent treatment. It does not establish efficacy when functional BSEP is absent or support routine PFIC treatment without specialist assessment.

For MDR3 deficiency, lipid nanoparticle delivery of human *ABCB4* mRNA restored phospholipid transport and improved liver injury in a mouse model of PFIC [94]. This is preclinical replacement therapy, distinct from the clinical use of UDCA in selected *ABCB4*-associated disease. Contemporary PFIC reviews describe additional pharmacological and gene-based approaches, but comparative human data remain limited [95].

The distribution of a transport inhibitor also matters. Intestine-restricted inhibition improved injury in a mouse model of sclerosing cholangitis [96], while systemic ASBT inactivation increased urinary bile acid elimination in obstructive cholestasis [97]. The systemic inhibitor A3907 subsequently showed effects in mouse models and tolerability with target engagement in healthy volunteers [98]. These findings do not demonstrate patient efficacy or replace drainage of an obstruction.

In a 62-patient PSC trial, the engineered FGF19 analogue NGM282, now known as aldafermin, reduced bile acid synthesis and some fibrosis-related biomarkers but did not meet the primary alkaline phosphatase endpoint [99]. Further suppression of synthesis may not benefit every disease stage. An observational study in two PSC cohorts associated already suppressed C4 with poorer transplant-free survival [100]. This finding supports testing stage-specific treatment hypotheses rather than assuming that lower synthesis is always preferable.

NTCP inhibition provides another example of indication-specific translation. Bulevirtide improved virological and biochemical responses in chronic hepatitis D and is authorized in the European Union for eligible patients with compensated liver disease [23,101]. This antiviral indication does not establish treatment of gallstones or postoperative cholestasis. TGR5 agonism, AQP8 modulation, and microbiota-directed treatment remain experimental for the surgical applications considered here [6,34,53,102].

Table 2 separates current indications from clinical-trial and preclinical evidence, with the surgical limitation stated for each approach.

Scheme 2 places these therapies within bile acid homeostasis and distinguishes established clinical uses from proposed mechanisms.

### 4.2. Implications for Biliary Surgery

The strongest surgical application is recognition of a specific disorder that anatomical treatment alone cannot resolve. Recurrent or intrahepatic stones, a suggestive family history, and cholestatic episodes can justify specialist assessment for LPAC or another inherited disorder [3]. A metabolic risk factor or an isolated genetic variant does not establish causality.

“Programmed cholecystectomy” is used here only as a hypothesis linking structured preoperative assessment to surgical selection and timing. The cited evidence does not validate a bile lithogenicity threshold, a fixed perioperative UDCA regimen, or a reduction in complications attributable to this approach. It should not alter established indications or delay necessary surgery outside a prospective study.

Common gallbladder stones and common bile duct stones require separate decisions. NICE recommends observation for asymptomatic stones in an otherwise normal gallbladder and biliary tree, but offers duct clearance and laparoscopic cholecystectomy for common bile duct stones, whether symptomatic or asymptomatic [105]. Molecular hypotheses cannot erase this anatomical distinction.

Endoscopic sphincterotomy and percutaneous cholecystostomy require established clinical indications. A transporter variant, dyslipidemia, or presumed bile lithogenicity does not justify prophylactic intervention. Coexisting gastrointestinal disease also requires its own assessment, since its symptoms may persist after cholecystectomy.

Postoperative cholestasis requires prompt assessment for retained stones, bile duct injury, stricture, infection, and nonobstructive hepatic causes. Molecular evaluation can follow when the phenotype remains unexplained or suggests inherited disease. It must not delay imaging, drainage, endoscopy, or reoperation when these are indicated. Scheme 3 sets out this sequence.

### 4.3. Diagnostic Uncertainty in Patients Without Typical Biliary Symptoms

In their chapter “Asymptomatic Cholecystitis: The Versatility of a Simple Clinical Question” in *Hepatobiliary Medicine and Surgery*, Muraviev and Ebrahimnezhad described 252 patients and reported frequent coexisting gastrointestinal and pancreatic abnormalities and postcholecystectomy symptoms [8]. The chapter uses “asymptomatic cholecystitis” for an atypical or clinically obscured presentation, which differs from the conventional definition of incidentally detected asymptomatic gallstones.

This observational experience raises a legitimate question about symptom attribution before surgery. It did not test C4, FGF19, or transporter genotyping as predictors of surgical benefit, and therefore cannot validate molecular selection for cholecystectomy. Gallbladder wall thickening, dyslipidemia, or pancreatic exocrine insufficiency alone does not establish a new molecular disease entity. Their value is in prompting a more precise differential diagnosis.

For research, patients with incidental stones, atypical symptoms, and established inflammatory or obstructive disease should be defined separately. A proposed biomarker must improve prediction beyond clinical history, laboratory tests, and imaging, and must identify an action that benefits the patient. Otherwise, more detailed biochemical description will not resolve the surgical dilemma.

### 4.4. Genetic Cholestasis and Non-Transplant Surgical Treatment

Genetic diagnosis can change the assessment of refractory cholestasis by identifying extrahepatic disease, malignancy risk, and the likelihood that sufficient bile secretion remains for interruption of enterohepatic circulation to help. Table 3 summarizes 13 genetic forms described within the expanding PFIC spectrum. Gene-based names are used because numbered classifications differ among publications.

Partial external biliary diversion (PEBD) diverts a portion of gallbladder bile to a cutaneous stoma. Partial internal biliary diversion (PIBD), including gallbladder-to-colon diversion, returns bile beyond the principal ileal uptake site without an external stoma. Ileal exclusion reduces contact with the terminal ileum. These operations aim to reduce recycling and relieve refractory symptoms in selected patients, usually before advanced liver disease makes transplantation the more appropriate option [3,103,104].

Earlier pooled PEBD evidence supported symptomatic benefit but drew on small, heterogeneous series [103]. A later synthesis of 25 studies included 424 children treated with PEBD, PIBD, or ileal exclusion and estimated pruritus resolution in 59.5% overall. Estimates were 57% for PEBD, 72% for PIBD, and 48% for ileal exclusion, with wide, overlapping confidence intervals [104]. The estimates describe heterogeneous observational cohorts and do not establish superiority of one procedure.

Genotype and response may be more informative than pooled averages. In the retrospective NAPPED cohort of 264 patients with BSEP deficiency, residual-function categories distinguished clinical courses and the association between diversion and native liver survival. A serum bile acid concentration below 102 μmol/L or a decrease of at least 75% after diversion predicted better long-term native liver survival [108]. These were post-treatment prognostic associations, not universal preoperative eligibility thresholds.

Table 4 compares the main non-transplant operations and the limitations relevant to their selection.

The decision should consider residual bile secretion, fibrosis, portal hypertension, growth, vitamin status, intestinal disease, and feasible long-term care. Trials of IBAT inhibitors and diversion series cannot be compared as if they enrolled the same patients. Medical treatment may defer or alter an operation in selected cases, but persistent symptoms or progressive liver disease require reassessment rather than indefinite escalation of antipruritic therapy [3,31,82].

### 4.5. Molecular Biomarkers and Surgical Decisions

Clinically actionable molecular assessment already includes phenotype-directed testing for inherited cholestasis and selected recurrent lithogenic disorders. A pathogenic or likely pathogenic result may establish the diagnosis, prompt genetic counseling and surveillance, or identify a treatment limitation such as absent functional BSEP [3,63,78,79]. A variant of uncertain significance does not independently justify a drug, diversion, or cholecystectomy. Standard imaging, liver tests, and fibrosis assessment remain part of clinical care rather than experimental molecular screening.

Bile metabolomics has potential diagnostic applications beyond inherited cholestasis. The BileMet study evaluated metabolic fingerprints to distinguish biliary tract cancer from benign biliary disease and reported an area under the receiver operating characteristic curve of 0.891 [109]. This is diagnostic discrimination in a specific study setting. It does not establish improved surgical outcomes, replace pathological assessment, or validate testing in asymptomatic gallstone disease.

C4 and FGF19 are most interpretable when measured for a defined question about bile acid synthesis or diarrhea [10]. C4 has also shown prognostic associations in PSC, but no threshold has been validated to select cholecystectomy or diagnose postoperative cholestasis [100]. Similarly, metabolomic signatures, transporter-expression assays, and experimental receptor readouts require validation for each intended use. Sampling conditions, external validation, and benefit from the resulting treatment decision matter more than discrimination alone.

### 4.6. Practical Interpretation for Surgeons

A focused clinical assessment follows from these distinctions.

Consider hepatology or genetics consultation for an early-onset, familial, recurrent, or otherwise atypical cholestatic phenotype. Interpret variants with the clinical findings and provide genetic counseling when appropriate [3].

Use C4 and FGF19 for a defined question within the settings in which they have been evaluated. Neither is a stand-alone test for postoperative cholestasis or the timing of cholecystectomy [10].

Select a therapy for its disease-specific indication and intended endpoint, using Table 2 to distinguish approved care from investigational treatment. For IBAT inhibitors, check labeling, residual BSEP function where relevant, gastrointestinal tolerance, liver tests, and fat-soluble vitamins [78,79,87].

Investigate a suspected mechanical complication promptly. An abnormal biochemical profile does not remove the need for anatomical assessment.

Discuss biliary diversion in a specialist multidisciplinary setting when inherited cholestatic disease remains symptomatic despite appropriate medical treatment. Assess the patient’s liver disease stage and the consequences of the proposed diversion [3,103].

### 4.7. Evidence Gaps and Research Priorities

The principal gap is whether molecular stratification improves decisions and outcomes in defined biliary surgical populations. Prospective cohorts should distinguish recurrent choledocholithiasis, inherited cholestasis, and patients with atypical symptoms. Candidate markers should be assessed against clinical and imaging predictors, with prespecified outcomes such as recurrent stones, repeat intervention, persistent symptoms, and quality of life. A prediction model requires independent validation before it is used to allocate treatment.

Interventional studies should test a specified drug or management strategy in a biologically defined population. Changes in bile acids or liver enzymes should be reported alongside patient outcomes, adverse effects, and treatment burden. The negative BOLD update also shows why postoperative disease-modification endpoints cannot be inferred from antipruritic efficacy [88]. Comparative studies of diversion and medical therapy are needed, while experimental perioperative strategies must not delay established treatment.

### 4.8. Limitations of the Review

This narrative review used selective retrieval and qualitative appraisal. It did not apply a registered protocol, duplicate screening, formal risk-of-bias assessment, or pooled effect estimation. Selection and language bias remain possible. Much of the evidence concerns rare pediatric disorders or nonsurgical populations, and small uncontrolled studies are vulnerable to confounding and selective publication. The authors’ published chapter provides clinical context rather than independent validation of the proposed framework. Regulatory status is time- and jurisdiction-dependent, and the BOLD update remains a sponsor report.

## 5. Conclusions

Bile biochemistry now contributes to biliary surgery beyond explaining mechanisms. It can inform disease-specific diagnosis, targeted treatment, and patient stratification. The principal contribution of this review is the integration of molecular bile acid biology with surgical decision-making.

Inherited transporter defects and selected recurrent lithogenic phenotypes provide the clearest examples. In these settings, molecular assessment can identify a defined disorder, explain recurrent symptoms after technically successful surgery, and direct patients to specialist treatment, genetic counseling, and long-term surveillance. For selected patients, this extends management beyond correction of anatomy and supports a diagnosis-specific therapeutic strategy.

IBAT inhibitors such as maralixibat, odevixibat, and linerixibat have established antipruritic efficacy in defined cholestatic populations. UDCA remains an established treatment for PBC and is used in selected *ABCB4*-associated disorders. Interventions targeting bile acid pathways can therefore provide symptom relief and disease-specific care. The preliminary negative BOLD report in biliary atresia shows that molecular efficacy must be evaluated within the disease, endpoint, and surgical context studied.

The evidence is organized into three levels encompassing mechanism, disease-specific therapy, and surgical outcome. This distinction limits unwarranted extrapolation of molecular findings to perioperative practice and identifies priorities for prospective studies. It also provides a framework for testing how bile acid biology can inform evidence-based biliary surgery.

In practice, this framework helps define relevant patient groups. Molecular information may identify patients who need specialist assessment rather than routine cholecystectomy, those who may benefit from targeted medical therapy, and those for whom anatomical intervention remains the priority. It therefore supports an integrated approach in which molecular and anatomical findings are considered together.

Prospective studies must determine whether molecular stratification improves outcomes beyond conventional clinical and imaging assessment. They should enroll clearly defined populations, use prespecified endpoints, and link testing to actionable treatment decisions. Positive results would support bile biochemistry as both a diagnostic adjunct and a therapeutic target in biliary surgery.

The available evidence does not support replacing established surgical indications with molecular testing. Bile biochemistry already informs the diagnosis and treatment of selected biliary disorders, while the remaining uncertainties define testable priorities for research. Future biliary care may integrate anatomical and molecular assessment when each addresses a defined clinical question and is supported by evidence.

## Figures and Tables

**Scheme 1:**
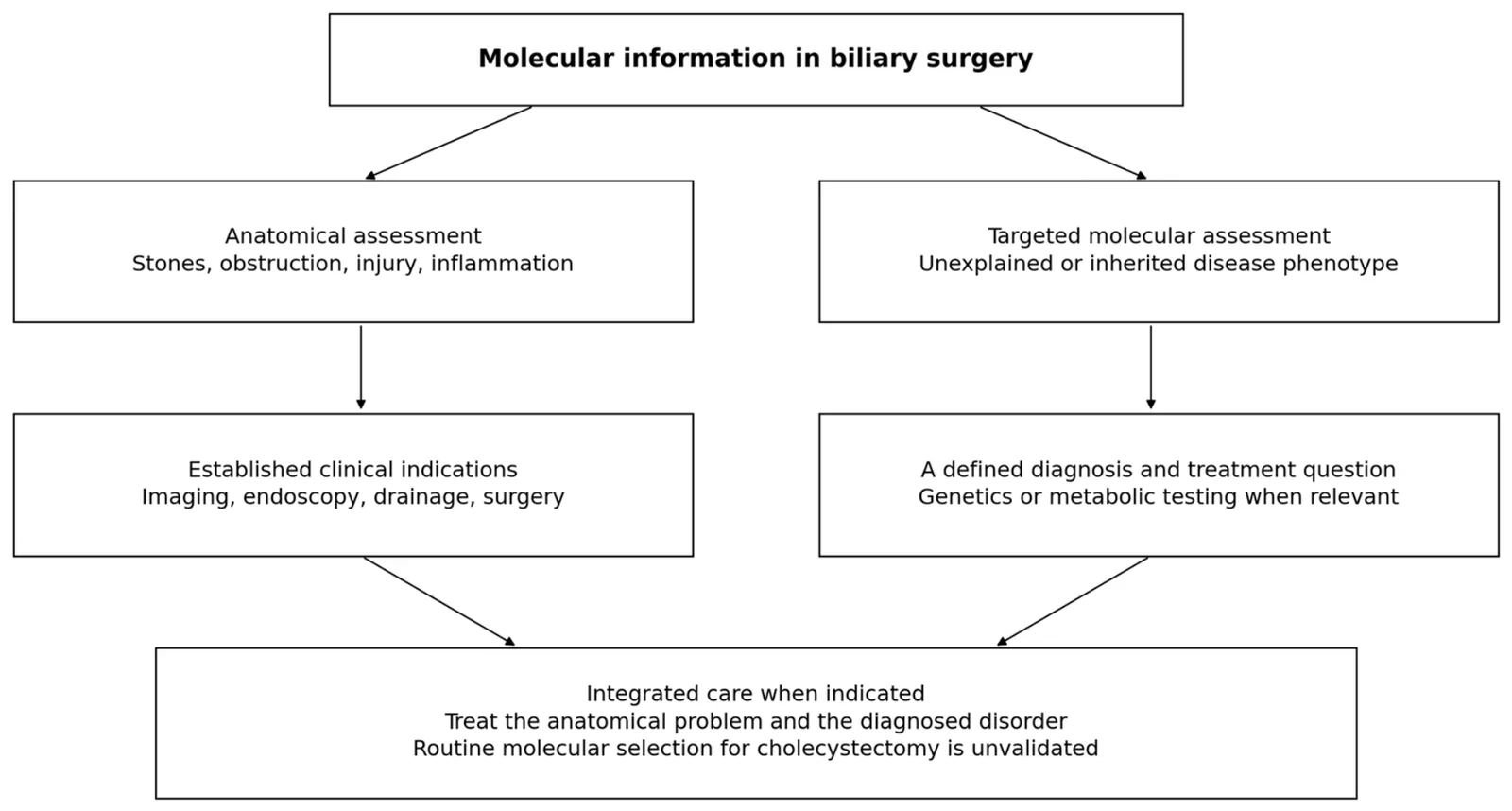
Molecular information in biliary surgical decisions. Testing is directed by a defined clinical question. Molecular profiling in patients described as having asymptomatic cholecystitis remains a research application, and the expected benefit is not a demonstrated reduction in complications.

**Scheme 2:**
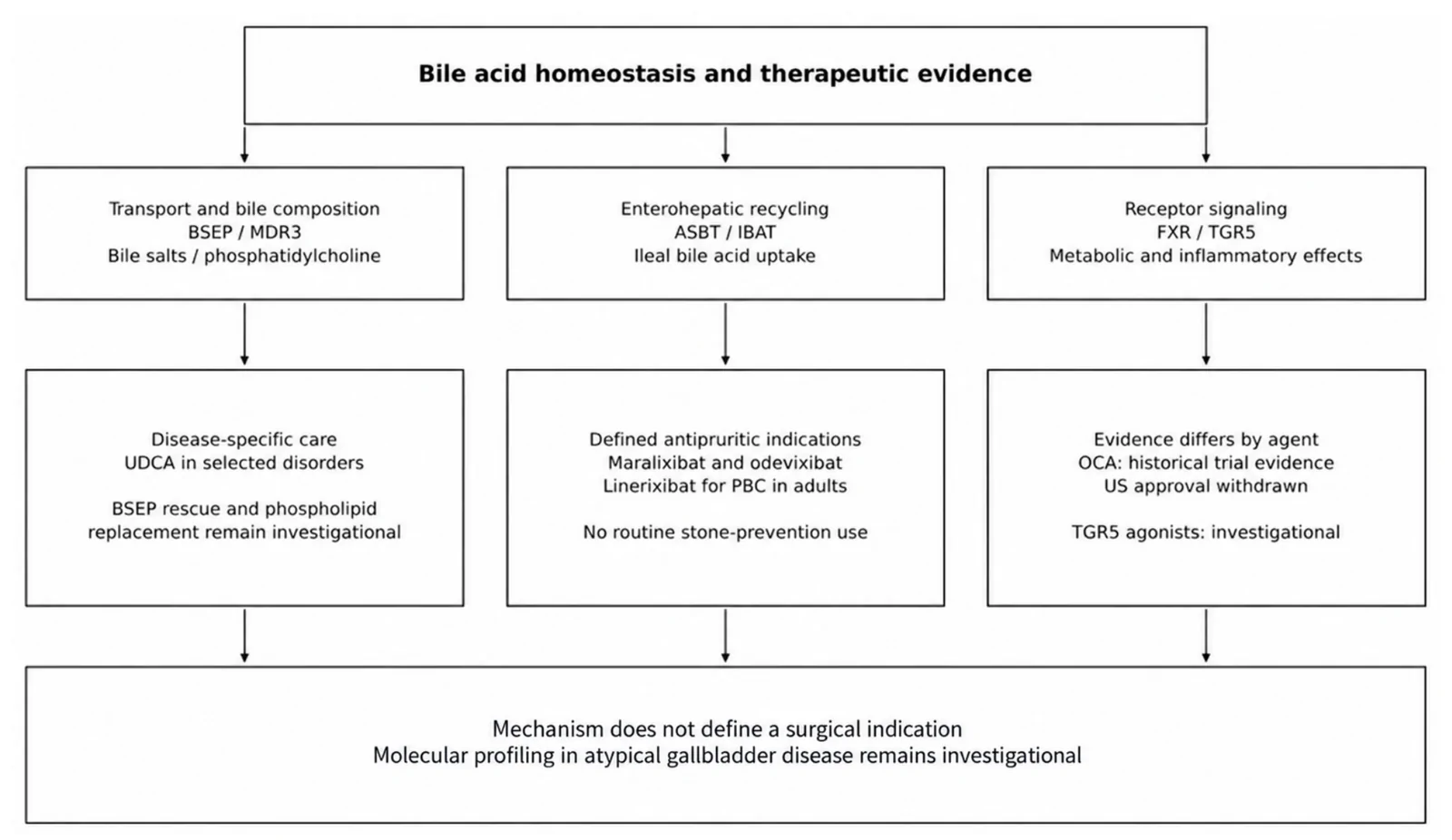
Therapeutic targets in bile acid homeostasis. Clinical indications and experimental approaches are distinguished. Regulatory statements refer to the United States unless otherwise specified and were checked on 20 September 2026. Symptom relief does not establish benefit for gallstone recurrence or perioperative outcomes. OCA, obeticholic acid.

**Scheme 3:**
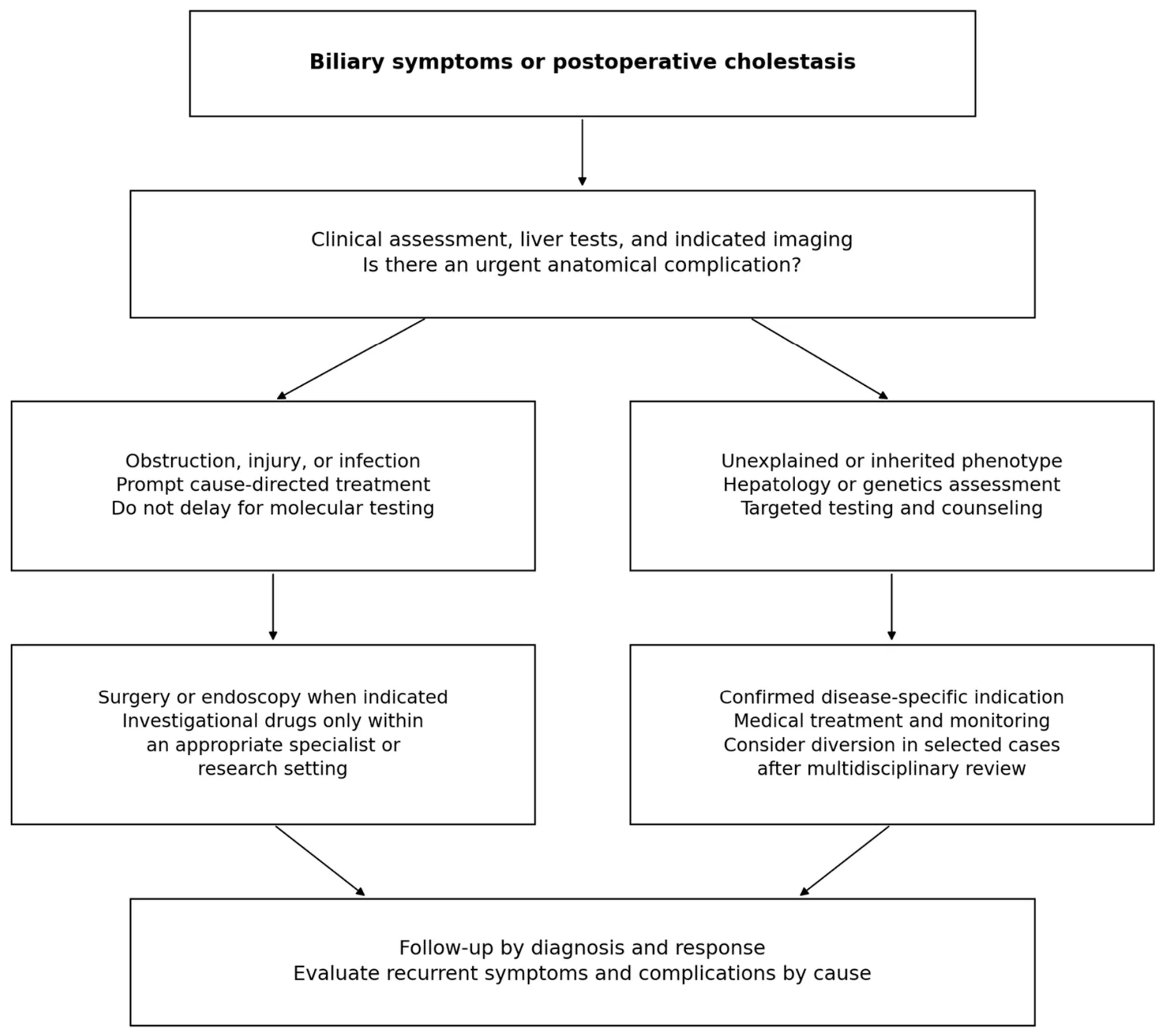
Clinical assessment before molecular treatment in biliary care. Urgent anatomical complications take priority. Biliary diversion requires specialist discussion of genotype, disease stage, and treatment response. Genetic counseling and phenotype-based follow-up are appropriate when inherited cholestasis is suspected. This conceptual framework is not a validated decision rule.

**Table 1:** Molecular components of bile acid homeostasis and their clinical interpretation.

Protein (Gene)	Location and Principal Function	Disease Association or Clinical Interpretation
CYP7A1 (*CYP7A1*)	Hepatic initiation of the classical synthetic pathway.	Rare deficiency was linked to hypercholesterolemia and gallstones in a family study [9].
CYP27A1 (*CYP27A1*)	Mitochondrial sterol oxidation and alternative-pathway initiation.	Deficiency causes cerebrotendinous xanthomatosis [11].
CYP7B1 (*CYP7B1*)	Oxysterol 7α-hydroxylation in the alternative pathway.	Deficiency can cause neonatal cholestatic liver disease [13].
BAAT (*BAAT*)	Hepatic conjugation with glycine or taurine.	Conjugation changes solubility and intestinal handling of bile acids [1,15].
NTCP (*SLC10A1*)	Sinusoidal bile acid uptake. Also a viral entry receptor.	Deficiency can cause hypercholanemia. Bulevirtide targets NTCP in hepatitis D [21,23].
OATP1B1 (*SLCO1B1*)	Sinusoidal uptake of organic anions and bilirubin conjugates.	OATP-C and SLC21A6 are historical aliases, not separate transporters [39].
BSEP (*ABCB11*)	Canalicular bile salt export.	Inherited cholestasis. Residual function influences phenotype and treatment selection [3].
MDR3 (*ABCB4*)	Canalicular phosphatidylcholine translocation.	PFIC, LPAC, and adult cholestatic disease. Distinct from MDR1 (*ABCB1*) [36,37].
FIC1 (*ATP8B1*)	Membrane phospholipid asymmetry.	PFIC with possible diarrhea, pancreatic disease, and other extrahepatic manifestations [3,32].
MRP2 (*ABCC2*)	Canalicular export of bilirubin conjugates and organic anions.	Deficiency causes Dubin–Johnson syndrome [40].
MRP3/MRP4 (*ABCC3*/*ABCC4*)	Basolateral efflux from hepatocytes.	Adaptive export during cholestasis. No established direct perioperative treatment [19,50].
AQP8 (*AQP8*)	Hepatocyte water transport.	Human expression associations and experimental steatosis findings. No therapeutic patient data [33,34].
ASBT/IBAT (*SLC10A2*)	Apical uptake in ileum and other epithelia.	Loss of function causes primary bile acid malabsorption. Drug effects depend on tissue exposure [7,59].
OSTα/β (*SLC51A*/*SLC51B*)	Basolateral export in enterocytes and hepatocytes.	Rare deficiencies cause intestinal bile acid malabsorption with cholestatic features [60,61].
FXR (*NR1H4*)	Nuclear regulation of synthesis, transport, and intestinal FGF19.	Biallelic deficiency causes severe inherited cholestasis. Drug benefit is disease-specific [3,62].
TGR5 (*GPBAR1*)	Membrane receptor in biliary, immune, and other cells.	Inflammatory and metabolic effects. Hepatobiliary agonist treatment remains investigational [52,53].
FGF19/FGFR4	Ileal hormone signaling through hepatic FGFR4 and β-Klotho.	Feedback suppression of synthesis. Therapeutic and tumor-associated effects require separate appraisal [1,56].

Human gene symbols are italicized and protein names are upright. PFIC, progressive familial intrahepatic cholestasis. LPAC, low-phospholipid-associated cholelithiasis. ASBT, apical sodium-dependent bile acid transporter. IBAT, ileal bile acid transporter. FXR, farnesoid X receptor. Other protein abbreviations are defined in the text.

**Table 2:** Therapies targeting bile acid pathways, their evidence, and relevance to biliary surgery.

Target	Agent or Approach	Current Indication or Status	Evidence and Principal Endpoint	Relevance to Biliary Surgery
Bile acid pool	UDCA	Established in PBC. Specialist use in LPAC and selected *ABCB4* disease.	Disease-specific clinical evidence and guidance [3,16].	Treat the diagnosed disorder. No universal perioperative regimen.
FXR	Obeticholic acid	Historical clinical therapy. US approval withdrawn in 2025.	FLINT histological activity in NASH. Regulatory withdrawal [65,67].	No demonstrated biliary surgical benefit.
FXR	Cilofexor and tropifexor	Investigational for the settings discussed.	Tropifexor phase 2 NASH biomarker effects. PRIMIS phase 3 PSC fibrosis endpoint negative [68,69].	Target engagement does not establish disease modification.
PPARδ	Seladelpar	US-approved for adult PBC with inadequate UDCA response or intolerance.	Phase 3 biochemical response and itch improvement. Accelerated approval [70,72].	PBC-directed care. No established surgical outcome benefit.
PPARα/δ	Elafibranor	US-approved for adult PBC with inadequate UDCA response or intolerance.	Phase 3 biochemical response. Key itch endpoint not significant. Accelerated approval [71,73].	Biochemical efficacy cannot be assumed to prevent surgical complications.
IBAT	Maralixibat	US antipruritic indications in ALGS and PFIC, with age and formulation limits.	Randomized antipruritic efficacy, external controls, and small practice series [75,76,78,80,81,82].	Can inform care before or after diversion. No proven stone-prevention effect.
IBAT	Odevixibat	US antipruritic indications in PFIC and ALGS, with age limits.	Phase 3 pruritus and bile acid responses, with additional practice data. BOLD postoperative survival endpoint negative in sponsor report [77,79,83,88].	Pruritus benefit does not establish improved survival after Kasai surgery.
IBAT	Linerixibat	US-approved for cholestatic pruritus in adults with PBC.	GLISTEN phase 3 demonstrated itch improvement [86,87].	Symptom treatment. No established perioperative indication.
IBAT	Elobixibat	Constipation treatment in Japan.	Clinical constipation evidence [89,90].	Different indication. No established biliary surgical role.
BSEP trafficking	4-Phenylbutyrate	Off-label or investigational in selected BSEP defects.	In vitro rescue and a four-patient uncontrolled study [91,93].	Requires a potentially rescuable variant and specialist assessment.
MDR3	*ABCB4* mRNA	Preclinical.	Improved phospholipid secretion and injury in PFIC mice [94].	No demonstrated efficacy in patients.
Systemic ASBT	A3907	Investigational.	Mouse efficacy and healthy-volunteer target engagement [98].	Patient and perioperative efficacy remain unproven.
FGF19 feedback	Aldafermin (NGM282)	Investigational in PSC.	Phase 2 primary ALP endpoint negative despite biomarker changes [99].	No validated surgical application.
NTCP	Bulevirtide	EU-authorized antiviral treatment for eligible chronic HDV infection.	Phase 3 virological and biochemical responses [23,101].	Antiviral indication, not cholestasis or stone prophylaxis.
Bile acid recycling	Biliary diversion	Selected inherited cholestatic disease.	Observational series and pooled analyses [103,104].	Pruritus relief must be balanced against procedure burden and disease stage.
TGR5, AQP8, microbiota	Receptor, water-transport, or microbial interventions	Experimental in the surgical settings reviewed.	Mechanistic, associative, and predominantly preclinical evidence [6,34,53,102].	No established perioperative regimen.

Evidence categories describe study design and are not formal certainty ratings. Regulatory information was checked on 20 September 2026. Approval depends on jurisdiction, disease, age, formulation, and liver disease stage. PBC, primary biliary cholangitis. PSC, primary sclerosing cholangitis. ALGS, Alagille syndrome. PFIC, progressive familial intrahepatic cholestasis. LPAC, low-phospholipid-associated cholelithiasis. HDV, hepatitis D virus. ALP, alkaline phosphatase. PPAR, peroxisome proliferator-activated receptor.

**Table 3:** Genetic diversity within the PFIC spectrum and implications for specialist management.

Gene and Protein	Principal Defect	Phenotype Relevant to Management	Implication and Evidence Limit
*ATP8B1* (FIC1)	Membrane phospholipid asymmetry.	Usually low/normal GGT cholestasis. Diarrhea and other extrahepatic disease may persist after transplantation.	Consider nutritional and intestinal consequences when comparing IBAT inhibition, diversion, and transplantation [3,32].
*ABCB11* (BSEP)	Canalicular bile salt export.	Usually low/normal GGT cholestasis. Severe deficiency carries early liver malignancy risk.	Residual function affects response. Specific variants associated with absent BSEP limit IBAT use [3,78,79].
*ABCB4* (MDR3)	Phospholipid secretion.	Usually high GGT. Childhood PFIC or adult LPAC and cholestatic phenotypes.	UDCA and management of lithogenic disease are phenotype dependent. Evidence for diversion is less established [3,37].
*TJP2* (TJP2)	Tight junction integrity.	Low/normal GGT cholestasis, sometimes rapid progression and liver malignancy.	Early specialist assessment and surveillance. Limited genotype-specific evidence for medical treatment or diversion [3,30].
*NR1H4* (FXR)	Bile acid sensing and transcriptional regulation.	Severe early cholestasis, often with coagulopathy.	Urgent assessment of hepatic function. Response to FXR agonism cannot be assumed in receptor deficiency [3,62].
*SLC51A* (OSTα)	Basolateral intestinal bile acid export.	Chronic diarrhea, malabsorption, and cholestatic liver disease.	Nutritional assessment is central. Evidence for further interruption of recycling is sparse [30,60].
*USP53* (USP53)	Tight junction-associated function.	Low/normal GGT cholestasis with variable severity. Hearing loss has been reported.	Assess extrahepatic findings and response individually. Small series dominate the evidence [30,32].
*KIF12* (KIF12)	Intracellular transport and hepatocyte polarity.	Usually high GGT cholestasis with variable onset and progression.	No established genotype-specific therapy. Stage and phenotype guide decisions [30,31].
*ZFYVE19* (ZFYVE19)	Ciliary organization and epithelial function.	High GGT cholestasis, ductal abnormalities, and possible portal hypertension.	Assess ductal anatomy and fibrosis. Therapeutic evidence remains limited [30,31].
*MYO5B* (myosin Vb)	Apical protein trafficking.	Low/normal GGT cholestasis, with or without microvillus inclusion disease.	Intestinal disease alters nutritional and surgical planning. Outcomes cannot be generalized across variants [30,32].
*SEMA7A* (semaphorin 7A)	Canalicular architecture and transporter localization.	Rare low/normal GGT cholestatic phenotype.	Few reported families. No established genotype-specific treatment [30,106].
*VPS33B* (VPS33B)	Vesicular trafficking and epithelial polarity.	Spectrum from liver-predominant cholestasis to arthrogryposis–renal dysfunction–cholestasis syndrome.	Renal disease, bleeding risk, and other systemic features affect procedural planning [30,32].
*PSKH1* (PSKH1)	Ciliary and epithelial function.	Recently described hepatorenal ciliopathy with cholestasis.	Assess renal involvement. Clinical evidence is limited to rare families and functional studies [31,107].

GGT patterns are typical rather than diagnostic. Phenotype and variant classification remain essential, especially for recently described genes. Related disorders, including *SLC51B* deficiency, can overlap this spectrum. The table does not imply equally established treatment evidence for all 13 forms. GGT, gamma-glutamyl transferase. Other abbreviations are defined in the text.

**Table 4:** Non-transplant operations for selected inherited cholestatic disorders.

Procedure	Anatomical Approach	Clinical Evidence	Selection and Treatment Burden
PEBD	Gallbladder-to-skin conduit diverts part of the bile externally.	Largest observational experience. Pooled pruritus resolution about 57% in one comparative review [104].	Requires an external stoma. Fluid and electrolyte loss, stoma complications, and care burden affect suitability. Response varies with genotype [3,108].
PIBD, including gallbladder-to-colon diversion	Bile is diverted internally to bowel beyond the principal ileal absorption site.	Pooled pruritus resolution about 72%, with wide uncertainty and selected cohorts [104].	Avoids a skin stoma. Diarrhea and procedure-specific intestinal complications remain possible. Lower pooled transplant rates cannot establish superiority.
Ileal exclusion	Terminal ileum is bypassed to reduce active reabsorption.	Smaller evidence base. Pooled pruritus resolution about 48%, with wide uncertainty [104].	Avoids external diversion but can worsen diarrhea and malabsorption. Durability and comparative benefit remain uncertain.

Percentages refer to pruritus resolution in one meta-analysis and should not be interpreted as individual probabilities of treatment success. The procedure groups differed in genotype, selection, and follow-up. No randomized comparison establishes a preferred operation or equivalence with IBAT inhibitors.

## Data Availability

No new data were created or analyzed in this study. Data sharing is not applicable to this article.
